# Supplementary figures and images for: MaSMG7-Mediated Degradation of MaERF12 Facilitates Fusarium oxysporum f. sp. cubense Tropical Race 4 Infection in Musa acuminata
Source: Int J Mol Sci. 2024 Mar 18;25(6):3420. doi: 10.3390/ijms25063420 (PMC10970408; doi:10.3390/ijms25063420)

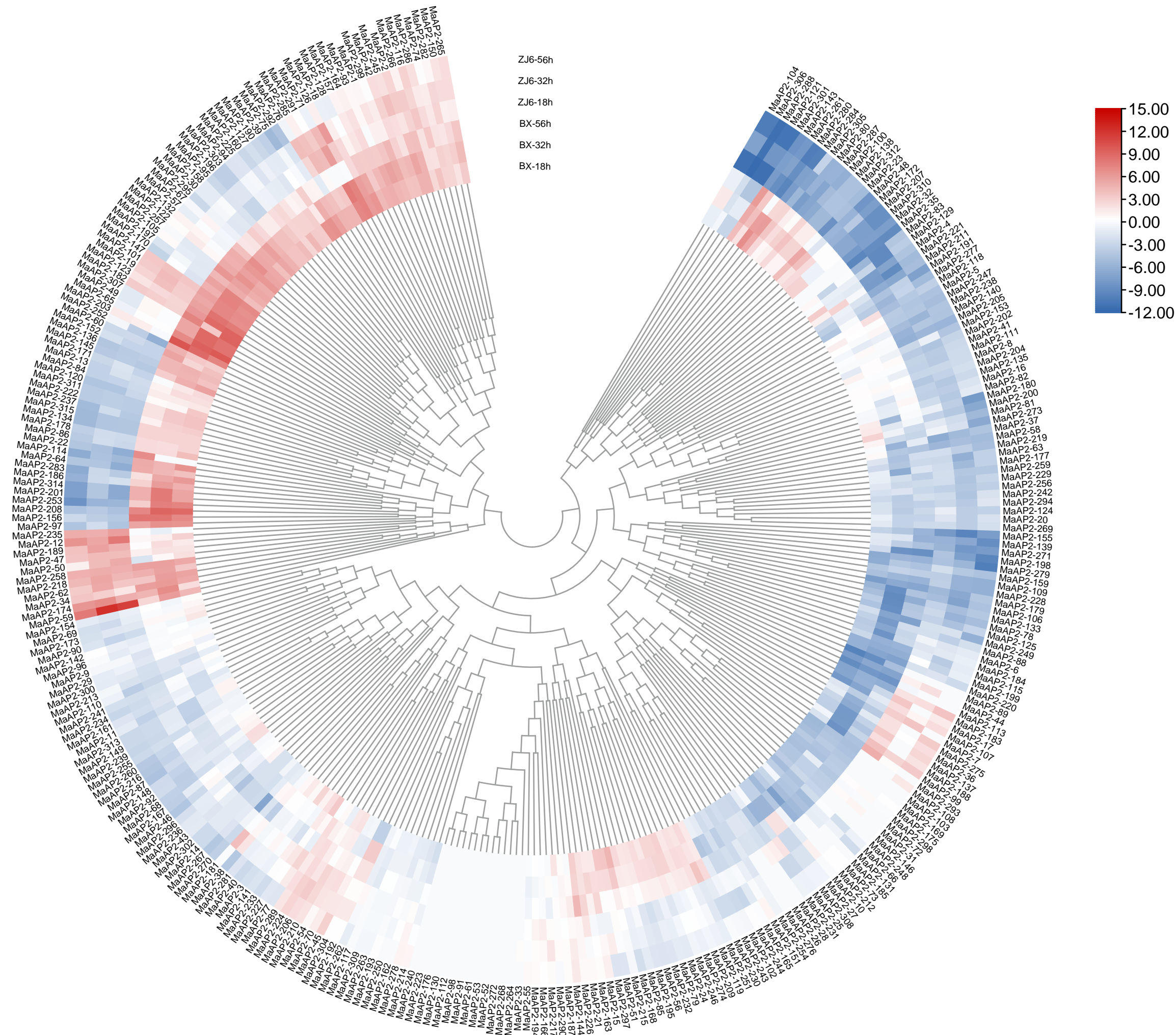

Supplement: Supplementary file 1 [file ijms-25-03420-s001.zip › Figure S1. Identification of AP2-ERF family.pdf]
